# The Smart-Insole Dataset: Gait Analysis Using Wearable Sensors with a Focus on Elderly and Parkinson’s Patients

**DOI:** 10.3390/s21082821

**Published:** 2021-04-16

**Authors:** Chariklia Chatzaki, Vasileios Skaramagkas, Nikolaos Tachos, Georgios Christodoulakis, Evangelia Maniadi, Zinovia Kefalopoulou, Dimitrios I. Fotiadis, Manolis Tsiknakis

**Affiliations:** 1 Biomedical Informatics and eHealth Laboratory, Department of Electrical and Computer Engineering, Hellenic Mediterranean University, Estavromenos, 71004 Heraklion, Greece; emaniadi@hmu.gr (E.M.); tsiknaki@hmu.gr (M.T.); 2 Computational BioMedicine Laboratory, Institute of Computer Science, Foundation for Research and Technology—Hellas, Vassilika Vouton, 71110 Heraklion, Greece; vskaramag@ics.forth.gr (V.S.); gxristod@ics.forth.gr (G.C.); 3 Unit of Medical Technology and Intelligent Information Systems, Department of Materials Science and Engineering, University of Ioannina, 45110 Ioannina, Greece; ntachos@uoi.gr (N.T.); fotiadis@cs.uoi.gr (D.I.F.); 4 Department of Biomedical Research, Institute of Molecular Biology and Biotechnology, Foundation for Research and Technology—Hellas, 45110 Ioannina, Greece; 5 Neurology Department, Patras University Hospital, 26404 Patra, Greece; zkefalopoulou@gmail.com

**Keywords:** gait analysis, Parkinson’s disease, insoles, pressure sensors, dataset

## Abstract

Gait analysis is crucial for the detection and management of various neurological and musculoskeletal disorders. The identification of gait events is valuable for enhancing gait analysis, developing accurate monitoring systems, and evaluating treatments for pathological gait. The aim of this work is to introduce the Smart-Insole Dataset to be used for the development and evaluation of computational methods focusing on gait analysis. Towards this objective, temporal and spatial characteristics of gait have been estimated as the first insight of pathology. The Smart-Insole dataset includes data derived from pressure sensor insoles, while 29 participants (healthy adults, elderly, Parkinson’s disease patients) performed two different sets of tests: The Walk Straight and Turn test, and a modified version of the Timed Up and Go test. A neurologist specialized in movement disorders evaluated the performance of the participants by rating four items of the MDS-Unified Parkinson’s Disease Rating Scale. The annotation of the dataset was performed by a team of experienced computer scientists, manually and using a gait event detection algorithm. The results evidence the discrimination between the different groups, and the verification of established assumptions regarding gait characteristics of the elderly and patients suffering from Parkinson’s disease.

## 1. Introduction

Gait is a rhythmic, periodic movement that requires coordination, balance, and synchronization, activated by the proper functioning of the central and peripheral (musculoskeletal) nervous system. Movement is powered by the ground reaction forces (GRF) applied to the body through its contact with the ground. The physical and psychological state of a person significantly differentiates the gait characteristics, creating a unique pattern for each person [1]. However, several typical patterns of gait can be detected that relate to normal, nonpathological gait. Accurate gait analysis is crucial for a variety of systems that support clinical experts in the diagnosis and management of patients that present pathological gait. Even more, it can provide evidence for the recognition of falls, which are common in older people [2].

**Figure 1:**
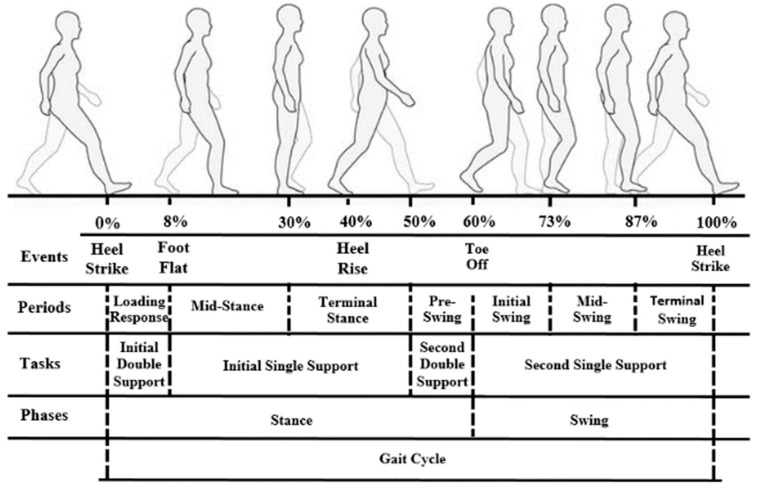
The gait cycle: events, periods, tasks, and phases. (Figure designed based on [6,11,12,13]).

Το analyze gait, the gait cycle is employed, which is identical to stride and includes two successive steps. The phases describing a gait cycle are: (a) the stance phase that occupies 60% of the gait cycle and (b) the swing phase that occupies 40% of the gait cycle [3,4,5]. The stance phase begins with the first contact of the foot with the ground and ends when the same foot leaves the ground, while the swing phase begins with the foot to leave the ground and ends with the next contact of the same foot with the ground. A more detailed breakdown of stance and swing phases into periods, namely: initial contact, loading response, mid-stance, terminal stance, pre-swing, mid-swing, and terminal swing, has been proposed in the literature in order to achieve a better understanding of gait [6,7]. For the analysis of a gait cycle, the recognition of the characteristic events of gait that describe the position/contact of the foot (heel and toe), with respect to the ground, is needed. In a normal gait cycle, the sequence of events that takes place is the Heel Strike, which indicates the beginning of stance, followed by the Foot Flat and Heel Rise, until the Toe Off event appears, which indicates the beginning of the swing phase, and ends with the next *Heel Strike*. The sequence of the characteristic gait events and the correlation of the different subdivisions of gait phases (events, periods, and phases) can be seen in Figure 1. Typically, force plates are used as sensing systems for measuring the GRF during gait, which provide accurate analysis. Nevertheless, force plates are relatively high-priced, require expert operation and are limited to laboratory settings [8]. The use of wearable sensors has the advantage of transferring gait analysis out of the laboratory in daily life. Towards this direction, sensor insoles for measuring foot pressure is one of the current state-of-the-art technologies used for gait analysis since each gait event can be described with a pressure pattern, and thus, phases and gait patterns can be analyzed [9]. Alternatively, inertial measurement units (IMUs) and electromyography (EMG) signals are widely exploited to power algorithms for discriminating the gait phases and patterns [10]. Combinations of the aforementioned sensory systems are also used [6]. In this work, pressure sensor insoles are exploited for the generation of the Smart-Insole dataset, as an appropriate solution for gait analysis in real-life settings, with the focus on elderly and Parkinson’s disease patients.

## 2. The Smart-Insole Dataset

Smart-Insole is a publicly available dataset (available for download from https://bmi.hmu.gr (accessed on 14 April 2021) focused on gait analysis, which includes data from a pair of pressure sensors insoles where 29 participants from 3 different groups of interest (healthy adults, elderly, Parkinson’s disease patients), performed two sets of tests. The protocol of the Smart-Insole study has received ethical approval from the Hellenic Mediterranean University Research Ethics Committee (Approval number: 9/01.04.2020). The dataset’s generation details regarding the equipment, the measurement protocol, the undertaking tests, the participant’s details, and the following annotation process are described in Section 2.1, Section 2.2 and Section 2.3.

### 2.1. Data Acquisition Details

**Figure 2:**
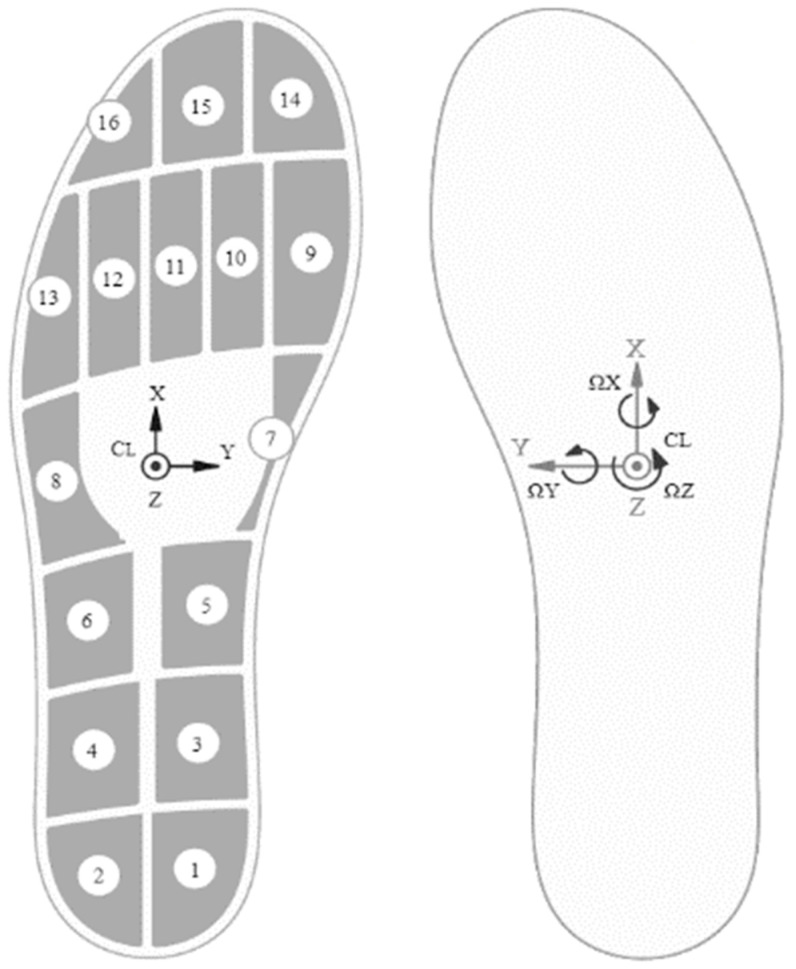
The Moticon SCIENCE pressure sensor insole (model insole 3). Reproduced with permission from Moticon ReGo AG [14].

For the development of the Smart-Insole Dataset, the Moticon SCIENCE [14] pressure sensor insole was selected (Figure 2). This choice was based on the following facts: (a) the power supply, the storage, and the data transmission unit are integrated into the insole; (b) the system has a sufficient number (16) of pressure sensors; (c) it includes a 6-Axis Inertial Measurement Unit (IMU) sensor for acceleration and angular rate data. The insole system has been validated in terms of functionality and accuracy of the data it provides [15,16,17]. For the needs of the recordings, a lightweight and flexible pair of shoes was purchased in which the insole was fitted.

The sampling rate was set at 100 Hz. The generated file for each recording includes 51 features in total, 25 values for the left and 25 values for the right leg, plus the timestamp:

1. The timestamp (ms)
2. The pressure from 1 to 16 sensors (N/cm^2^)
3. The acceleration in the x,y,z axes (g)
4. The angular rate in ω_x_, ω_y_, ω_z_ (dps)
5. The computed center of pressure in the x,y coordinates (−0.5…+0.5 (related to insole length/width))
6. The computed by Moticon, total force (N)

### 2.2. Measurement Protocol Details

Several measurement protocols have been proposed for the evaluation of gait characteristics, postural stability, as well as risk of falling for people who have a normal or abnormal gait. Most of these protocols include trials with a number of steps in a straight path and at different speeds, walking on a sloped surface and on stairs. In the case of people with an abnormal gait, and especially for patients with Parkinson’s disease, the following three tests have been mostly performed: (1) the Timed Up and Go test (TUG test) [18,19,20,21,22], where the participants rise from a sitting position, walk a 3 m distance, turn, walk back and sit on a chair; (2) walking in a corridor with obstacles, [23,24,25] and; (3) the Dual-Task test, where the participants walk and at the same time undertake a second process, such as an arithmetic operation, conversation, transferring an object, etc. [26,27,28].

The Smart-Insole’s measurement protocol was developed having in mind the previously described findings and the need for: (a) simplicity, so that it is easily understandable by all groups of participants (healthy adults, elderly, Parkinson’s disease patients); (b) safety, so that there is no physical exhaustion or even risk of falling, referring mainly to the elderly and Parkinson’s disease patients, and; (c) completeness of the experimental procedures, so that valuable data are recorded for the evaluation of a complete gait analysis system. Items from the part III “Motor examination” of the Movement Disorder Society (MDS)–sponsored revision of the Unified Parkinson’s Disease Rating Scale (UPDRS) [29] has been used for the clinical assessment of motor signs of Parkinson’s disease and as guidelines for the experimental protocol. Taking all these into consideration, the protocol for the generation of the Smart-Insole dataset includes the Walking Straight and Turn test and a modified version of the Timed Up and Go test, which are described in detail in Section 2.2.1 and Section 2.2.2. During the recordings, two action cameras were set along the route (one at the end and one at the middle of the route). The cameras were recording only the lower body part, i.e., the legs of the participants, so that the verification of the recorded raw data during the annotation process would be possible. However, it is worth mentioning that the public version of the dataset includes only the anonymized data containing the raw values from the insole sensors (for which participants have signed consent forms on making them publicly available in the scientific community) and the annotated version of the data as described in Section 2.4.

#### 2.2.1. The Walk Straight and Turn Test

**Figure 3:**
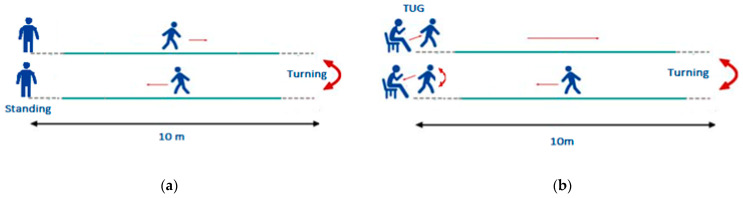
Left figure (**a**) The Walking Straight Test, and right figure (**b**) The Timed Up and Go test.

In the Walk Straight and Turn (WST) test, participants were requested to walk in a straight route for 10 m starting from an upright position (standing). At the end of the 10-m route, they turned 180° and returned to the starting position (Figure 3a). The WST test was organized in line with the description of the item-3.10 “Gait” of the MDS-UPDRS. From a clinical point of view, it provides valuable information for stride amplitude and speed, heel strike, and turning, while it also contributes to the rating of items 3.11 “Freezing of Gait” and 3.13 “Posture”. Participants were asked to complete the tests as a normal and continuous set of movements. The test was repeated two times and at three different walking speeds, slow, normal, and fast, as perceived by each participant. Different speeds of walking have been studied for their impact on gait characteristics [30] and on differencing gait between Parkinson’s disease patients with mild and moderate severity [31]. For the recordings, the start and endpoints of the route were clearly marked on the floor.

#### 2.2.2. The modified Timed Up and Go Test

In the modified Timed Up and Go (TUG) test, participants were instructed to rise from the chair (if possible, without any support of the upper limbs) naturally (without any intended stopping pose) and immediately started walking in a straight path for 10 m. At the end of the 10-m route, they made an 180° turn and returned to the sitting position (Figure 3b). In the typical form of TUG test, the walking distance is 3 m and the participants are rated with a completion time score which indicates their level of physical mobility [18]. In our work, the MDS-UPDRS has been used as a guideline for the clinical examination of motor signs of Parkinson’s Disease. Thus, in the deployed protocol, we included a modified version of the TUG test to obtain data from its initial phase, i.e., the getting up phase, which align with the 3.9-item “Arising from chair” of the MDS-UPDRS, but we extended the walking distance to 10 m to obtain more data during walking. The test was repeated twice.

### 2.3. Dataset Participants

The first release of the Smart-Insole dataset includes recordings from 29 participants who were categorized into three groups, as shown in Table 1. The first group is the control group, in which healthy adults aged between 20–59 years were included. The second group includes elderly citizens, in which people above the age of 60 years were included. For these two groups, volunteers having musculoskeletal or neurological diseases that could affect their gait or balance were excluded from participation. The third group relates to Parkinson’s disease patients, irrespective of their age. All participants with Parkinson’s disease had received their medication as normally scheduled. For the participants that would find it hard to complete all tests, the number and repetitions were adjusted so that they felt comfortable.

A Neurologist specialized in movement disorders evaluated the performance of the participants by rating four items from part III “Motor examination” of the MDS-UPDRS [32], i.e., item 3.9 “Arising from chair”, item-3.10 “Gait”, item-3.11 “Freezing of gait”, and item-3.14 “Global spontaneity of movement”, that match with the measurement protocol followed. In so doing, the neurologist has carefully examined the video recordings obtained by the two cameras (one at the end and one at the middle of the 10 m-route) used during the recordings. Previous works evidence the feasibility of remote assessment of PD patients even via videoconference sessions. Specifically, part III “Motor examination” of the MDS-UPDRS (excluding item-3.3 “Rigidity” and item-3.12 “Postural Stability”, which require an in-person assessment), has been evaluated for the assessment of motor signs of PD in several studies [33,34,35,36,37]. The MDS-UPDRS rating for each participant is shown in Table 2.

### 2.4. Smart-Insole Dataset Annotation

The labeling of the recorded data was performed following a two-level annotation process. In the first level of annotation, data were described as activities of daily living (ADLs), following the labeling protocol of our previous work “MobiAct” [38], a benchmark dataset for activity and fall recognition. The first level of annotation of ADLs, included 12 different labels (Table 3), which described the sequence of activities undertaken by the participants in the tests. The second level of annotation was focused on labeling the characteristic events of a gait cycle (HES-FOF-HER-TOF-HES). For gait analysis, the labeling of events of both legs was mandatory. In a normal gait cycle, the described sequence starts with the one foot on Heel Strike, and the other foot on Toe Off. Consequently, in the Smart-Insole dataset the labels of four events of gait can be found for the left and right leg as described in Table 4.

For the annotation of the data, we followed a hybrid model of both manual and automated labeling. In each case, to reassure the reliability of data labeling, cross-checks with the signal alternation and the captured videos were performed. The annotation of ADLs was made purely manually, through inspection of signal alternation and the captured video regarding the timestamp.

**Figure 4:**
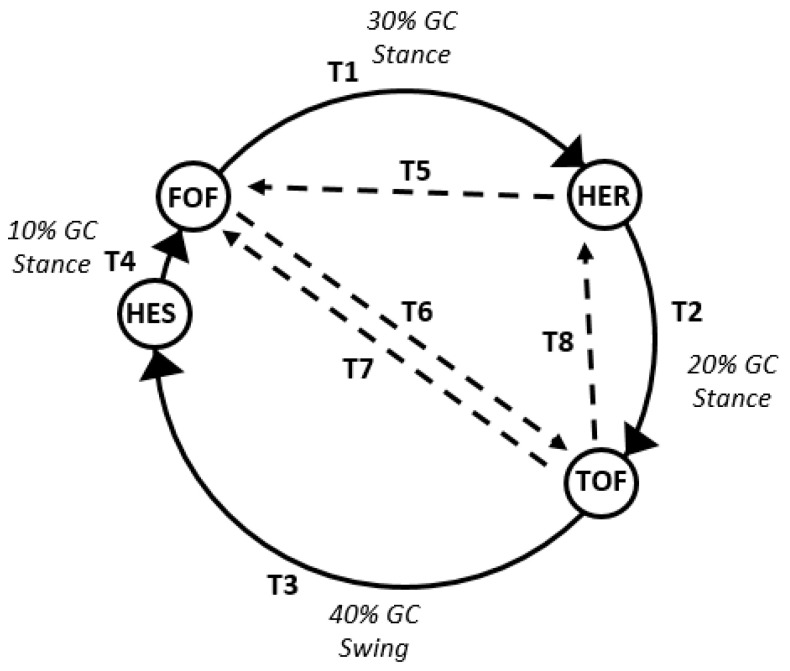
The core workflow of the developed gait event detection algorithm (based on [39] transition of states).

The automated annotation of gait events was made using a gait event detection algorithm that was developed for this purpose. The results of the algorithm were cross-validated using the signal and video data. The developed gait event detection algorithm was based on: (a) a modified version (Figure 4) of the core body of knowledge referring to transition between states, of the gait phase detection algorithm developed by Pappas et al. [39]; (b) the pressure threshold parameter (Equation (1)), for heel and toe of both left and right legs; (c) the heel and toe parameters, which store the average values of pressure sensors with respect to the timestamp inquest, and d) the previous state parameter, which holds the previous event of the normal flow of a gait cycle (GC) namely, HES-FOF-HER-TOF.

Initially, a pressure detection algorithm, based on Equation (1), was deployed to determine if the deployed pressure exceeded the threshold and, thus, the inquest area of the foot touching the ground. The pressure detection algorithm was based on the force detection algorithm, included in the timing analysis module (TAM) of the Tekscan software [40], in a similar manner as deployed in the work of Catalfamo et al. [41]. The only difference is that Catalfamo et al. used a force-based sensing system. In our work, the pressure factor was empirically adjusted, through a continuous test-and-check process, for each participant for the left and right leg separately. The assigned values for the pressure factor span from 0.04–0.12, while in the original work of Catalfamo et al. [36] it was set to 0.1. The min and max variables of Equation (1) are the minimum and maximum pressures of the recorded data.

(1) Pressure Threshold=min+(max−min)×Pressure Factor.

We considered that pressure sensors 1 and 2 represented the area of the heel (heel parameter), while sensors 14, 15, and 16, represented the pressure area of the toe (toe parameter), according to the arrangement of sensors shown in Figure 2. Employing more sensors (1–4 for heel and 9–16 for toe) did not affect the results. Thus, it was decided to use the minimum set of sensors.

The core workflow of the developed gait event detection algorithm is shown in Figure 4. It is based on the gait-phase detection methodology followed by Pappas et al. [39,42], who proposed seven transitions of activities between two gait events (Heel Off, Heel Strike) and two phases (Swing, Stance). In our work, we adopt these seven transitions (T1-T7) with the modification that we foresee them as transitions between the four gait events (heel strike, foot flat, heel rise, toe off) of a gait cycle. The sequence of events and its association with the transitions T1-T2-T3-T4, are shown in Figure 4. In the Smart-insole dataset, all tests begin with the participants in the standing or sitting positions and, thus, the gait begins from FOF, in consequence, the first transition T1 refers to the transition of FOF to HER event. Transitions T1, T2, T3, and T4 are expected to be observed in a normal gait cycle. The abnormalities that are most likely to appear, are covered by the transition T5, T6, T7 and T8 (added by our team). In the abnormal transition T5, the foot, instead of following a Toe Off event, and thus getting in to swing phase, moves to Foot Flat state from the Heel Rise, and retaining the stance; in the T6, Toe Off from Foot Flat is observed instead of Heel Rise; and in the T7, the foot is flat on the floor after Toe Off event instead of Heel Strike event. These abnormalities are most likely to appear in the gait of the elderly and in Parkinson’s disease patients since they could express uncertainty on the gait, lack of stability, and disorientation. The T8 transition relates to the abnormality of toe walking. Since the Heel Rise event describes a state of the foot where only the toe touches the ground, it can also be seen as a Toe On event. Therefore, the T8 transition describes a loop of Toe On and Toe Off events. Toe walking is mainly seen in children [43]. It has neither been observed in the Smart-Insole dataset nor is expected to be observed in the target population under study. It is, however, described for reasons of completeness. Data were labeled with the name of the event taking place until a new one is identified. If abnormalities are observed, then the previous event is also reported in the annotated version of the dataset. For both normal and abnormal transitions, the swing phase is considered to start when the Toe Off event occurs and to end when a new event takes place (T3, T7, T8), while in all other cases the stance phase prevails (T1, T2, T4, T5, and T6). The condition checks and the respective labels of the developed gait event detection algorithm for labeling are shown in Table 5.

## 3. Gait Analysis Methodology

The analysis of human body movement includes the notions of kinetics and kinematics. Kinetics describes the causes of movement and focuses on the forces (mass, acceleration), the torques, and the produced power of a movement. In contrast, kinematics focuses on the linear and angular description of the movement (changes in velocity position, displacement, and acceleration, over time). Therefore, gait analysis requires the quantified study of the force parameters, and the time and distance parameters, by calculating important characteristics (temporal and spatial, respectively).

The temporal characteristics can be categorized as purely temporal, phase temporal, or spatiotemporal. The phase-temporal characteristics have been normalized based on the duration of the gait cycle. The spatial characteristics have been calculated following an empirically driven methodology, based on the knowledge of the total distance of the route. For the estimation of the spatiotemporal characteristics data were divided into straight lines and turns, based on the analysis of video recordings and the respective timestamps. Straight lines’ data include recordings until the last step of straight walking, while Turns’ data include recordings from the first step indicating a change of direction (Toe Off event) until the final return step (Heel Strike event), of participants facing the return route. Therefore, three samples of recordings were analyzed for each participant’s record, two for straight-line walking and one during turning. The distance of the route for straight line walking corresponds to 10 m, ±1 step since some participants turned slightly before or after the marked 10 m aisle. Nevertheless, we claim that this does not have a major effect on the results of spatial characteristics, taking into consideration the range of the number of steps number (14.45 to 22.47), shown in Table 6. Performing a cross-check on our calculations (by adjusting ± 1 step) of the step length (as a key metric for spatial characteristics as given by Equation (13)), we can observe that the effect is limited to the second decimal place most of the time. The distance cover during turning corresponds to 1.5 m, as a mean value of a gait cycle’s length (Table 6), which is evidence for both spin-turn and step-turn strategies [44]. For the evaluation of the Smart-Insole dataset, and for the recognition of gait phases and events as a mandatory predecessor of gait pattern recognition, the following gait temporal and spatial characteristics were calculated:

- The *Step Time* (s), which is described as the time between two successive Heel Strikes of different foot.

(2) $$Step\;Τime=Heel\;Strike_{j+1}-Heel\;Strike_{j}.$$

- The *Stride Time* (s), which is equal to the time between two successive Heel Strikes of the same foot.

(3) $$Stride\;Τime=Heel\;Strike_{j+2}-Heel\;Strike_{j}$$

- The *Stance Time* (s, %), which describes the total time during a gait cycle where the foot is in contact with the ground. Specifically, it is described as the time where the heel of one foot, contacts the ground until the toe of the same foot leaves the ground.

(4) $$Stance\;Time=Toe\;Off_{j+1}-Heel\;Strike_{j},$$

(5) $$Stance\;Phase\left(\%\right)=\left(\frac{Stance\;Time}{Gait\;Cycle}\right)\times 100\%.$$

- The *Single Support Time* (s, %), which describes the time from the Toe Off of the one foot until the Heel Strike of the other foot.

(6) $$Single\;Support\;Time=\left(Heel\;Strike_{j}-Toe\;Off_{j-1}\right)+\left(Heel\;Strike_{j+1}-Toe\;Off_{j}\right),$$

(7) $$Single\;Support\;Phase\left(\%\right)=\left(\frac{Single\;Support\;Time}{Gait\;Cycle}\right)\times 100\%.$$

- The *Double Support Time* (s, %), which describes the time from the Heel Strike of the one foot until the Toe Off of the other foot.

(8) $$Double\;Support\;Time=\left(Toe\;Off_{j}-Heel\;Strike_{j}\right)+\left(Toe\;Off_{j-1}-Heel\;Strike_{j-1}\right),$$

(9) $$Double\;Support\;Phase\left(\%\right)=\left(\frac{Double\;Support\;Time}{Gait\;Cycle}\right)\times 100\%.$$

- The *Swing Time* (s, %), which describes the time from the Toe Off of the one foot until the Heel Strike of the same foot.

(10) $$Swing\;Time=Heel\;Strike_{j+1}-Toe\;Off_{j},$$

(11) $$Swing\;Phase\left(\%\right)=\left(\frac{Swing\;Time}{Gait\;Cycle}\right)\times 100\%.$$

- The *Gait Velocity* (m/s), which describes the displacement in the unit of time, is given by the ratio of the total distance to the total time, or by the ratio of the mean values of stride length to stride time.

(12) $$Gait\;Velocity=\frac{Stride\;Length}{Stride\;Time}.$$

- The *Step Length* (m) is calculated by dividing the total distance covered (20 m) to the total number of steps (Steps Number) which is specified as the number of Heel Strikes during gait.

(13) $$Step\;Lenght=\frac{Distance}{Steps\;Number}$$

- The *Stride Length* (m) is calculated by dividing the total distance covered (20 m) to the total number of strides (Strides Number).

(14) $$Stride\;Lenght=\frac{Distance}{Strides\;Number}.$$

- The *Step Frequency* (steps/min) also called cadence or walking rate, describes the number of steps in the unit of time. It is given by the ratio of the steps number to the time of gait, multiplied by 60 to be expressed in minutes.

(15) $$Step\;Frequency=\frac{Steps\;Number}{Time}\times 60.$$

- The *Walk Ratio* (mm/step/min) represents the relationship between the width (base of gait) and the frequency of steps and is given by the ratio of step length to Step Frequency.

(16) $$Walk\;Ratio=\frac{Step\;Length}{Step\;Frequency}.$$

## 4. Results

The experimental results of the temporal and spatial characteristics calculated, are shown in Table 6 for straight-line walking, and in Table 7 for turning. All results and related parameters correspond to mean values and their respective standard deviations, while the distance covered equals 10 m for straight lines and 1.5 m for turns. The results are presented according to the type of tests performed and the different groups of participants. The results for WST normal test and the TUG test could be grouped since both represent the self-selected normal pace of walking, and the parts that have been analyzed match. However, we present them separately for potential future comparison. From the Smart-Insole dataset, the participant of the Parkinson’s patient group with the code number PD008, was excluded from the calculations since he needed a walker to even stand in an upright position. For safety and comfort reasons, one elderly participant completed only one WST at high speed, while two of the Parkinson’s patients did not complete all the repetitions or tests. The number of recordings exploited for each group of participants and tests, can be found in Table 6 for straight-line walking and in Table 7 for turning. The reported results and standard deviations have been rounded to two decimal places, and thus, slight deviations may be found in cross-checks. Furthermore, the *p*-values of the estimated results are reported in Table 8 and Table 9, to examine the statistical significance of the discrimination between the different groups of participants.

**Figure 5:**
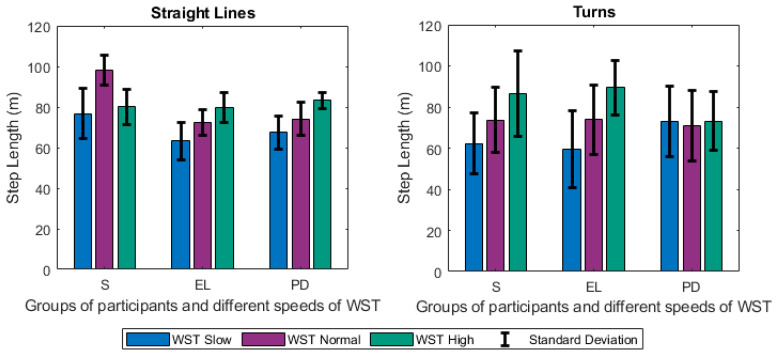
Changes of the Step Length (m) for Straight Lines and Turns (mean values along with standard deviations) between the different groups of participants (S, EL, PD) and the different speeds of the WST test (slow, normal, high).

**Figure 6:**
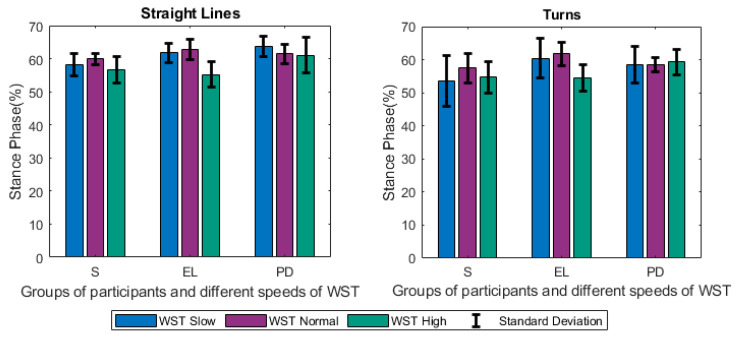
Changes of the Stance Phase (%) for Straight Lines and Turns (mean values along with standard deviations) between the different groups of participants (S, EL, PD) and the different speeds of the WST test (slow, normal, high).

**Figure 7:**
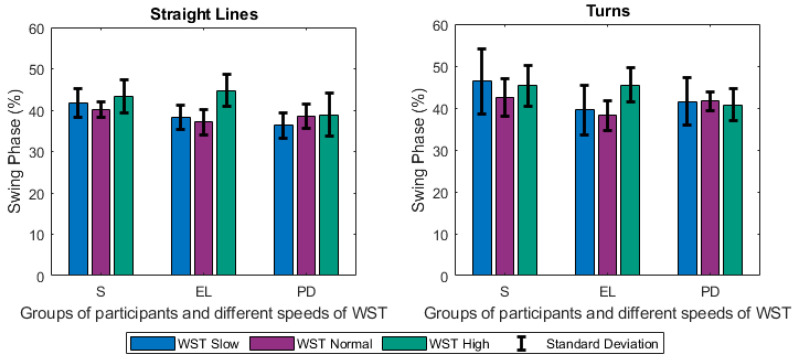
Changes of the Swing Phase (%) for Straight Lines and Turns (mean values along with standard deviations) between the different groups of participants (S, EL, PD) and the different speeds of the WST test (slow, normal, high).

**Figure 8:**
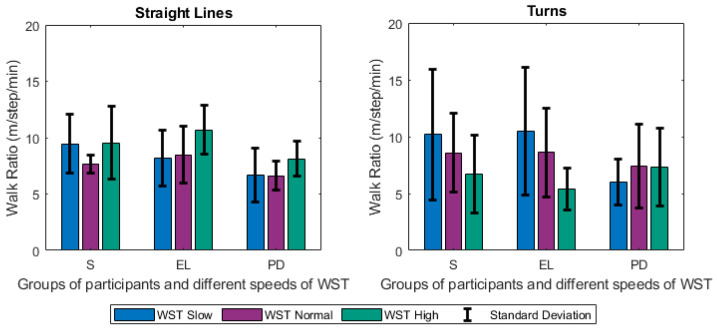
Changes of the Walk Ratio (m/step/min) for Straight Lines and Turns (mean values along with standard deviations) between the different groups of participants (S, EL, PD) and the different speeds of the WST test (slow, normal, high).

**Figure 9:**
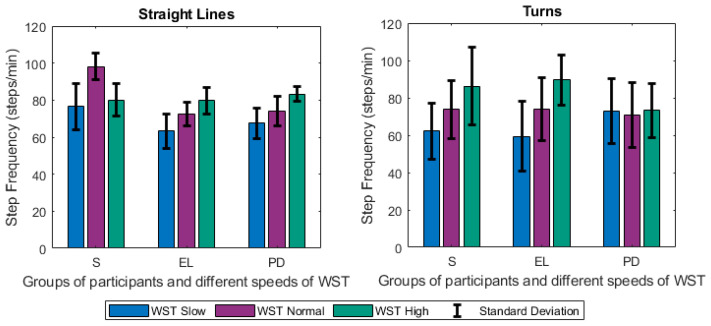
Changes of the Step Frequency (steps/min) for Straight Lines and Turns (mean values along with standard deviation) between the different groups of participants (S, EL, PD) and the different speeds of the WST test (slow, normal, high).

Focusing firstly on the different tests and groups, we notice that the different speeds of gait significantly affected the estimated gait characteristics. Changes were observed among others on the step length (Figure 5), the duration of stance (Figure 6), and swing (Figure 7) phases, and were mainly noted in the analysis of straight-line walking. Once someone increases his/her gait velocity, it is expected that the step length and the swing will increase, while the stance will decrease. Taking as baseline the WST normal, which represents a self-selected normal pace by participants, these changes are observed in all groups when moving to the *WST high* in straight-line walking, in which participants increased their pace of gait. It is worth mentioning that the mean values of the height of the participants (Table 1) differ only slightly and, thus, the effect of this parameter is only minimal. An important aspect to highlight is that % of stance and swing phases decrease and increase, respectively, during straight-line walking in the case of reduction of gait velocity (WST Slow). In this case, the step length also decreases (Figure 5, Figure 6 and Figure 7). The change can also be observed in the walking ratio for all groups. The ratio increases both when slowing down (WST Slow) and speeding up (WST high), as shown in Figure 8. These observations on slow walking are in line with previously published results [45,46]. Regarding the TUG test, increased values for the walk ratio, the step length, the step frequency, as well as, the stance time and phase were observed because of the participation of PD005. The specific patient exhibited a walking pattern of dragging his feet, something that affects the estimation of the respective metrics, and their standard deviations. The stride parameters are affected in a similar manner to the step parameters. The step frequency increases proportionally with the gait velocity (Figure 9), as expected. The duration of stance and swing phases (60%, 40% of the total gait cycle) were verified when examining the results of WST Normal and *TUG* test, which were recorded in normal velocity for the group of healthy adults (S), with ~61% and ~39% duration, respectively. Regarding the duration of the left and right steps, which for normal gait are expected to be equal, it was verified for the majority of the results on all groups. One should always keep in mind that: a) the ±0.1 differences are due to rounding of decimals, b) the differences that appeared for the S group, especially in the WST high test were due to the recording of one participant (S008) in which a highly disproportional gait was observed (0.3 s for the left and 0.6 s for the right step). This deviation, which is expected in pathological gait, is indeed apparent in the WST High test for the group of Parkinson’s patients.

These results reaffirm the importance of studying different velocities of gait for best revealing gait abnormalities. Furthermore, it is noted that the number of steps, the stance duration, and the double support duration increased for each test as we moved from healthy adults (normal values) to elderly (increased values) and finally to Parkinson’s disease patients. Finally, the low standard deviations of the results indicate that data are nicely clustered around the means. Although a low standard deviation is not necessarily required, it is evidence of the fact that all groups exhibited similar behaviors.

In demonstrating the ability of the estimated spatiotemporal characteristics to discriminate between the different groups of participants, when all three levels of speeds for the WST test and the TUG test are taken into consideration, statistical analysis has been performed by estimating the *p*-values of the results (Table 8, for Straight Lines, and Table 9 for Turns). The following combinations of participants’ groups were tested for discrimination for each test: (a) healthy adults (S) versus elderly (EL); (b) healthy adults (S) versus Parkinson’s disease patients (PD); and (c) elderly (EL) versus Parkinson’s disease patients (PD). The calculated probabilities show clear discrimination, when examining the results on straight-line walking, with statistically significant *p* < 0.01 for (a) the S-EL groups, for the WST slow, the WST normal, and the TUG tests; (b) the S-PD groups, for all tests; and (c) the EL-PD groups for the WST high test. Regarding the probabilities on turning data, we cannot state discrimination of the different groups; however, the *p*-values range in many cases from *p* < 0.01 to *p* = 0.05.

## 5. Discussion

Several datasets suitable for gait analysis, which have been created using sensor insoles [47,48], IMUs [30,49], or pressure and force plates [50,51], are publicly available. However, several concerns exist, with the most important one being the lack of evaluation and/or annotation of the data by specialized neurologists. A second shortcoming is the fact that only the Gait in Parkinson’s disease dataset [47] and the dataset proposed by Kluge et al. [30] include data from Parkinson’s disease patients. Although the Gait in Parkinson’s disease dataset includes a sufficient number (93) of participating Parkinson’s disease patients, it only includes recordings of their normal-pace walking. On the other hand, the dataset produced by Kluge et al. [30], includes recordings of Parkinson’s disease patients walking with different speeds but includes a very small number (4) of participants.

It is known and most studied for the elderly that different speeds of walking have an impact on the estimated results of spatial and temporal characteristics of gait [52,53]. Although limited work has been done in this domain focusing on Parkinson disease patients [30,54], it has been evidenced that short distance walking speed tests can be used to discriminate differences in gait function between persons with mild and moderate PD severity [31]. This evidence, together with the observations from our work, as described in Section 4, aligns with the suggestions of Wu et al. [45] to study the regressions between different gait speeds. These findings suggest that additional work is required for the effect of different levels of walking speed (slow, normal, and fast) on gait analysis.

Critical examination of the MDS-UPDRS ratings of the different groups, shown in Table 2, could lead to a conclusion of a ceiling effect, especially for the groups of elderly and healthy subjects. However, taking into consideration that the exclusion criteria of the protocol used for the groups of elderly and healthy subjects refers to no musculoskeletal or neurological diseases that could affect their gait or balance, this supports our decision to unify the groups from a clinical point of view. Taking a closer look at the groups of elderly and Parkinson’s disease patients, this could also be the case for some participants (EL002, EL007, PD002, PD004, and PD006). Nevertheless, these participants have slight gait impairment since they belong to the elderly population (Table 1 shows the average ages of 72 and 74, respectively) irrespective of whether they are Parkinson’s disease patients or not. These observations lead to the conclusion/suggestion that for applying computational methods for gait analysis with respect to the MDS-UPDRS, the grouping of participants data should be done not only by age differentiation but also taking their MDS-UPDRS rating into consideration.

These gaps and shortcomings of the available datasets are removed with the release of the Smart-Insole dataset, when used as a dataset for the evaluation of computational methods for detailed gait analysis (exploiting sufficient number of sensors) that can be applied in everyday life (wearable sensor insole), with the focus on elderly people and Parkinson’s patients (by adjusting the measurement protocol,) and with the reliability of data annotation provided by a specialized neurologist (rating of four MDS-UPDRS items). However, an increased number of participants would be required to thoroughly examine the gait characteristics of PD patients. A limitation of our study is the lack of a magnetometer, required for the accurate estimation of distance-related parameters. In the present study, these parameters have been approximated using the total distance (m) covered, as described in Section 3.

## 6. Conclusions

The presented Smart-Insole dataset, can be used for the detailed analysis of gait since, to our knowledge, it is the first public gait-related dataset in which the participant’s data have been evaluated by a neurologist specialized in movement disorders. It encompasses a fair number of participants of different groups; it is structured in a measurement protocol which is in line with the MDS-UPDRS; and it is developed with the use of wearable sensing equipment as an affordable solution that can be transferred in real life environments. The developed gait event detection algorithm has been double-cross-validated, empirically, by exploiting the recorded video data and, quantifiably by the calculation of the gait characteristics and the verification of established assumptions, as described in Section 4 (propositions of step and stride, stance and swing, left and right step, etc.). The experimental results show clear discrimination between the different groups of users, and the different set of tests performed with different gait speeds.

As this work, has been performed in the context of the Smart-Insole Project, it is the intention of the authors to extend and enrich the dataset: (a) with the use of the current Moticon sensor insole and with the use of a novel smart pressure sensors insole, which is under development in the context of the Smart-Insole project; (b) with the clinical evaluation of the participants by a specialized neurologist using the overall MDS-UPDRS; and (c) with an updated measurement protocol targeting on revealing important cardinal features of Parkinson’s disease patients. Finally, the next steps include the deployment of methods for gait pattern recognition and detection.

## Figures and Tables

**Table 1:** The details of Smart-Insole’s Dataset participants.

Group	No. of Recordings	Average Age (Years)	Age Span (Years)	Height (cm)	Weight (Kg)	Gender
Healthy adults (S)	13	38	20–58	176	81	2 Females, 11 Males
Elderly (EL)	9	74	60–85	173	80	All Males
PD patients (PD)	8	72	63–83	172	80	All Males

The public version of the Smart-Insole dataset does not include the data of PD001; thus, although eight participants of the “Parkinson” group are used in the results of this study, the public version of the dataset includes seven recordings.

**Table 2:** The rating of four MDS-UPDRS items [29], based on the analysis of participant’s videos.

Participant *	“MDS-UPDRS-3.9” Arising from Chair	“MDS-UPDRS-3.10” Gait	“MDS-UPDRS-3.11” Freezing of Gait	“MDS-UPDRS-3.14” Global Spontaneity of Movement
PD001	2	3	1	2
PD002	0	1	0	1
PD003	4	4	4	4
PD004	1	1	0	1
PD005	3	2	1	3
PD006	0	1	0	1
PD007	1	2	0	2
PD008	4	3	0	3
EL001	0	0	0	0
EL002	1	1	0	1
EL003	1	0	0	0
EL004	1	0	0	0
EL006	0	0	0	0
EL007	1	1	0	1
EL008	0	0	0	0
EL009	0	0	0	0
EL010	0	0	0	0
S001	0	0	0	0
S002	0	0	0	0
S003	0	0	0	0
S004	0	0	0	0
S005	0	1	0	0
S006	0	0	0	0
S007	0	0	0	0
S008	0	0	0	0
S009	0	0	0	0
S010	0	0	0	0
S011	0	0	0	0
S012	0	1	0	0
S013	0	0	0	0

* PDxxx refers to Parkinson’s patients, ELxxx refers to elderly, and Sxxx refers to healthy adults participating in the Smart-Insole dataset. The tag EL005 has not been used.

**Table 3:** Activities of daily living (ADLS): annotation of Smart-Insole dataset, Level 1.

Label	Activity	Description
STD	Standing	Standing with subtle movements
STE	Standing Eyes closed	Standing with eyes closed
WAL	Walking	Normal walking
WAS	Walking Slow	Walking in a slow rhythm
WAF	Walking Fast	Walking in a fast rhythm
SCH	Sit on chair	Sitting on a chair
CHU	Chair up	Getting up from a chair
SIT	Sitting	Sitting with subtle movements
TUR	Turning	Turning 180 degrees at a normal speed at the end of the 10 m aisle.
TUS	Turning Slow	Turning 180 degrees at a slow speed at the end of the 10 m aisle.
TUF	Turning Fast	Turning 180 degrees at a fast speed at the end of the 10 m aisle.

**Table 4:** Characteristic events of gait analysis: annotation of Smart-Insole dataset, Level 2.

Label	Activity	Description
HES	Heel Strike	Heel contacts the floor
HER	Heel Rise	Heel rises of the floor
TOF	Toe Off	Toe leaves the floor
FOF	Foot Flat	Foot is flat on the floor; both heel and toe contact the floor.

**Table 5:** The condition checks of the developed gait event detection algorithm for labeling.

Label	Condition Check
HES	(Heel>Force Threshold Heel)∧(Toe≤Force Threshold Toe)∧(Previous State=TOF)
FOF	(Heel>Force Threshold Heel)∧(Toe>Force Threshold Toe)∧(Previous State=HES)
HER	(Heel≤Force Threshold Heel)∧(Toe>Force Threshold Toe)∧(Previous State=FOF)
TOF	(Heel≤Force Threshold Heel)∧(Toe≤Force Threshold Toe)∧(Previous State=HER)
FOF (from HER)	(Heel>Force Threshold Heel)∧(Toe>Force Threshold Toe)∧(Previous State=HER)
TOF (from FOF)	(Heel≤Force Threshold Heel)∧(Toe≤Force Threshold Toe)∧(Previous State=FOF)
FOF (from TOF)	(Heel>Force Threshold Heel)∧(Toe>Force Threshold Toe)∧(Previous State=FOF)
HER (from TOF)	(Heel≤Force Threshold Heel)∧(Toe>Force Threshold Toe)∧(Previous State=TOF)

**Table 6:** Results on Straight Lines (Mean values along with Standard Deviations) of temporal and spatial characteristics using the Smart-Insole Dataset.

Type of Test	WST Slow			WST Normal			WST High			TUG		
Group of Participants	S	EL	PD	S	EL	PD	S	EL	PD	S	EL	PD
Number of Recordings	52	36	14	52	36	14	52	34	14	52	36	22
Left Step Duration (s)	0.75 ± 0.13	0.76 ± 0.17	0.70 ± 0.10	0.55 ± 0.05	0.58 ± 0.07	0.62 ± 0.15	0.48 ± 0.10	0.51 ± 0.12	0.51 ± 0.11	0.55 ± 0.15	0.44 ± 0.14	0.54 ± 0.09
Right Step Duration (s)	0.75 ± 0.14	0.77 ± 0.15	0.73 ± 0.14	0.57 ± 0.06	0.58 ± 0.08	0.58 ± 0.06	0.54 ± 0.09	0.49 ± 0.12	0.53 ± 0.07	0.57 ± 0.10	0.62 ± 0.14	0.63 ± 0.18
Step Duration (s)	0.75 ± 0.13	0.76 ± 0.14	0.72 ± 0.09	0.56 ± 0.04	0.58 ± 0.05	0.60 ± 0.09	0.51 ± 0.06	0.50 ± 0.05	0.52 ± 0.06	0.57 ± 0.08	0.54 ± 0.03	0.58 ± 0.10
Stride Duration (s)	1.50 ± 0.26	1.54 ± 0.28	1.44 ± 0.20	1.13 ± 0.08	1.18 ± 0.10	1.20 ± 0.20	1.02 ± 0.13	1.00 ± 0.09	1.04 ± 0.12	1.12 ± 0.16	1.07 ± 0.08	1.18 ± 0.23
Steps Number	15.70 ± 2.38	22.47 ± 5.60	22.28 ± 5.30	14.45 ± 1.15	20.11 ± 5.80	19.57 ± 4.07	12.75 ± 1.48	14.91 ± 1.86	17.07 ± 3.60	12.78 ± 1.99	17.61 ± 3.90	17.30 ± 5.00
Single Support Time (s)	1.26 ± 0.11	1.27 ± 0.24	1.16 ± 0.24	0.91 ± 0.10	0.87 ± 0.08	0.98 ± 0.24	0.84 ± 0.12	0.83 ± 0.08	0.77 ± 0.12	0.89 ± 0.13	0.83 ± 0.05	0.91 ± 0.23
Double Support Time (s)	0.27 ± 0.13	0.36 ± 0.16	0.39 ± 0.16	0.22 ± 0.08	0.30 ± 0.10	0.29 ± 0.09	0.18 ± 0.07	0.17 ± 0.11	0.27 ± 0.09	0.22 ± 0.09	0.24 ± 0.09	0.25 ± 0.09
Stance Time (s)	0.86 ± 0.17	0.93 ± 0.19	0.89 ± 0.16	0.67 ± 0.06	0.72 ± 0.09	0.72 ± 0.15	0.59 ± 0.11	0.56 ± 0.08	0.65 ± 0.09	0.63 ± 0.08	0.63 ± 0.01	0.77 ± 0.43
Swing Time (s)	0.61 ± 0.10	0.57 ± 0.08	0.50 ± 0.04	0.45 ± 0.04	0.42 ± 0.03	0.45 ± 0.04	0.45 ± 0.04	0.45 ± 0.02	0.41 ± 0.07	0.43 ± 0.04	0.40 ± 0.05	0.40 ± 0.04
Single Support (%)	86.49 ± 10.26	83.45 ± 8.77	81.94 ± 11.88	80.64 ± 7.15	76.62 ± 8.57	82.72 ± 8.78	81.54 ± 8.02	82.74 ± 11.05	73.27 ± 8.17	82.99 ± 9.07	79.91 ± 8.10	78.82 ± 8.17
Double Support (%)	16.76 ± 7.86	21.91 ± 7.57	20.92 ± 10.34	19.82 ± 6.45	25.90 ± 7.39	24.06 ± 4.10	17.94 ± 5.40	16.04 ± 9.12	25.97 ± 9.00	20.15 ± 7.45	23.25 ± 6.88	21.99 ± 7.72
Stance Phase (%)	58.26 ± 3.37	61.74 ± 2.81	63.69 ± 3.04	59.88 ± 1.79	62.86 ± 3.10	61.48 ± 2.95	56.57 ± 4.01	55.21 ± 3.85	61.09 ± 5.25	59.21 ± 2.78	61.21 ± 2.79	63.84 ± 6.01
Swing Phase (%)	41.74 ± 3.37	38.26 ± 2.81	36.31 ± 3.04	40.12 ± 1.79	37.14 ± 3.10	38.52 ± 2.95	43.45 ± 4.01	44.79 ± 3.85	38.91 ± 5.25	40.79 ± 2.78	38.79 ± 2.79	36.16 ± 6.01
Gait Velocity (m/s)	0.99 ± 0.20	0.77 ± 0.26	0.71 ± 0.20	1.39 ± 0.17	1.21 ± 0.34	1.00 ± 0.29	1.79 ± 0.28	1.72 ± 0.35	1.31 ± 0.35	1.67 ± 0.37	1.47 ± 0.36	1.25 ± 0.45
Step Length (m)	0.70 ± 0.11	0.56 ± 0.14	0.50 ± 0.12	0.75 ± 0.06	0.68 ± 0.16	0.56 ± 0.11	0.87 ± 0.14	0.82 ± 0.12	0.65 ± 0.12	0.88 ± 0.18	0.75 ± 0.16	0.70 ± 0.35
Stride Length (m)	1.44 ± 0.25	1.14 ± 0.29	1.00 ± 0.26	1.56 ± 0.15	1.40 ± 0.34	1.16 ± 0.23	1.82 ± 0.36	1.70 ± 0.27	1.33 ± 0.27	1.86 ± 0.43	1.57 ± 0.37	1.52 ± 0.92
Step Frequency (steps/min)	76.67 ± 12.43	63.21 ± 9.11	67.59 ± 8.14	98.18 ± 7.17	72.47 ± 6.24	74.19 ± 8.09	80.00 ± 8.76	79.68 ± 7.23	83.21 ± 4.01	100.67 ± 12.24	71.45 ± 8.14	65.18 ± 19.87
Walk Ratio (mm/step/min)	9.46 ± 2.65	8.16 ± 2.48	6.69 ± 2.41	7.68 ± 0.78	8.49 ± 2.56	6.63 ± 1.27	9.55 ± 3.21	10.68 ± 2.17	8.12 ± 1.53	9.07 ± 3.37	13.14 ± 3.46	26.02 ± 53.65

**Table 7:** Results on Turns (mean values along with standard deviations) of temporal and spatial characteristics using the Smart-Insole dataset.

Type of Test	WST Slow			WST Normal			WST High			TUG		
Group of Participants	S	EL	PD	S	EL	PD	S	EL	PD	S	EL	PD
Number of Recordings	26	18	7	26	18	7	26	17	7	26	18	11
Left Step Duration (s)	0.77 ± 0.23	0.80 ± 0.25	0.77 ± 0.28	0.59 ± 0.16	0.60 ± 0.09	0.62 ± 0.12	0.52 ± 0.16	0.56 ± 0.17	0.58 ± 0.13	0.61 ± 0.17	0.45 ± 0.25	0.65 ± 0.24
Right Step Duration (s)	0.79 ± 0.30	0.88 ± 0.38	0.70 ± 0.48	0.59 ± 0.21	0.63 ± 0.16	0.50 ± 0.18	0.58 ± 0.13	0.50 ± 0.17	0.49 ± 0.20	0.58 ± 0.11	0.63 ± 0.17	0.81 ± 0.79
Step Duration (s)	0.78 ± 0.19	0.85 ± 0.22	0.69 ± 0.22	0.60 ± 0.11	0.61 ± 0.01	0.63 ± 0.15	0.55 ± 0.11	0.52 ± 0.08	0.60 ± 0.16	0.59 ± 0.09	0.56 ± 0.08	0.72 ± 0.48
Stride Duration (s)	1.56 ± 0.41	1.69 ± 0.44	1.49 ± 0.52	1.19 ± 0.27	1.24 ± 0.19	1.13 ± 0.14	1.11 ± 0.22	1.06 ± 0.20	1.08 ± 0.21	1.19 ± 0.19	1.09 ± 0.19	1.46 ± 0.97
Steps Number	3.75 ± 0.79	4.10 ± 1.12	4.70 ± 0.48	3.68 ± 0.62	3.77 ± 0.80	4.42 ± 1.13	4.07 ± 0.79	4.35 ± 0.60	4.16 ± 0.75	3.80 ± 0.78	4.11 ± 0.83	4.25 ± 0.75
Single Support Time (s)	0.97 ± 0.22	1.17 ± 0.27	1.01 ± 0.17	0.88 ± 0.14	0.88 ± 0.20	0.92 ± 0.23	0.79 ± 0.14	0.86 ± 0.12	0.81 ± 0.19	0.89 ± 0.10	0.76 ± 0.17	0.98 ± 0.49
Double Support Time (s)	0.30 ± 0.24	0.46 ± 0.26	0.36 ± 0.28	0.33 ± 0.14	0.35 ± 0.13	0.32 ± 0.11	0.24 ± 0.14	0.23 ± 0.13	0.33 ± 0.18	0.26 ± 0.11	0.37 ± 0.15	0.34 ± 0.23
Stance Time (s)	0.73 ± 0.25	0.91 ± 0.31	0.73 ± 0.23	0.62 ± 0.12	0.69 ± 0.11	0.63 ± 0.10	0.55 ± 0.11	0.55 ± 0.09	0.63 ± 0.09	0.60 ± 0.10	0.64 ± 0.10	0.82 ± 0.61
Swing Time (s)	0.61 ± 0.11	0.57 ± 0.08	0.50 ± 0.04	0.45 ± 0.04	0.42 ± 0.03	0.45 ± 0.04	0.45 ± 0.04	0.45 ± 0.02	0.43 ± 0.04	0.44 ± 0.05	0.40 ± 0.05	0.43 ± 0.13
Single Support (%)	73.54 ± 14.96	74.66 ± 12.17	81.75 ± 13.24	79.89 ± 12.67	72.70 ± 12.13	76.16 ± 12.52	79.29 ± 12.23	80.46 ± 8.04	71.16 ± 13.90	80.35 ± 8.07	68.75 ± 13.52	74.40 ± 9.75
Double Support (%)	18.19 ± 13.64	26.40 ± 11.75	17.89 ± 15.19	25.65 ± 12.97	31.78 ± 10.60	23.73 ± 9.56	21.05 ± 12.32	21.61 ± 10.34	25.71 ± 18.01	21.11 ± 8.48	33.84 ± 12.29	28.88 ± 10.07
Stance Phase (%)	53.62 ± 7.72	60.47 ± 5.97	58.48 ± 5.64	57.43 ± 4.51	61.80 ± 3.57	58.41 ± 2.21	54.65 ± 4.89	54.52 ± 4.00	59.26 ± 3.74	57.49 ± 3.87	61.12 ± 4.56	62.86 ± 11.57
Swing Phase (%)	46.38 ± 7.72	39.53 ± 5.97	41.52 ± 5.64	42.57 ± 4.51	38.20 ± 3.57	41.59 ± 2.21	45.35 ± 4.89	45.48 ± 4.00	40.74 ± 3.74	42.51 ± 3.87	38.88 ± 4.56	37.14 ± 11.57
Gait Velocity (m/s)	0.93 ± 0.38	0.78 ± 0.27	0.67 ± 0.20	1.28 ± 0.40	1.16 ± 0.25	1.14 ± 0.33	1.19 ± 0.32	1.17 ± 0.50	1.21 ± 0.51	1.12 ± 0.29	1.24 ± 0.45	0.94 ± 0.43
Step Length (m)	0.57 ± 0.18	0.54 ± 0.17	0.41 ± 0.06	0.59 ± 0.14	0.58 ± 0.15	0.48 ± 0.15	0.52 ± 0.14	0.46 ± 0.10	0.50 ± 0.14	0.57 ± 0.16	0.52 ± 0.14	0.49 ± 0.14
Stride Length (m)	1.35 ± 0.35	1.26 ± 0.38	0.96 ± 0.37	1.44 ± 0.21	1.42 ± 0.24	1.29 ± 0.37	1.30 ± 0.34	1.19 ± 0.38	1.25 ± 0.39	1.33 ± 0.32	1.29 ± 0.35	1.19 ± 0.39
Step Frequency (steps/min)	62.28 ± 15.00	59.32 ± 18.72	72.95 ± 17.26	73.72 ± 15.67	73.99 ± 16.91	70.87 ± 17.20	86.30 ± 20.78	89.49 ± 13.29	73.22 ± 14.27	74.53 ± 12.37	79.61 ± 12.67	72.67 ± 17.52
Walk Ratio (mm/step/min)	10.18 ± 5.71	10.48 ± 5.62	6.00 ± 2.03	8.59 ± 3.44	8.60 ± 3.88	7.38 ± 3.67	6.74 ± 3.42	5.38 ± 1.83	7.33 ± 3.42	8.05 ± 3.28	6.93 ± 3.18	8.17 ± 7.00

**Table 8:** *p*-values of the estimated results for Straight Lines with statistically significant *p* < 0.01 for three combinations of participants’ groups (S/EL, S/PD, EL/PD) and for each different test.

Type of Test	WST Slow			WST Normal			WST High			TUG		
Groups Compared	S/EL	S/PD	EL/PD	S/EL	S/PD	EL/PD	S/EL	S/PD	EL/PD	S/EL	S/PD	EL/PD
Recordings per Group	52/36	52/14	36/14	52/36	52/14	36/14	52/36	52/14	36/14	52/36	52/14	36/14
Left Step Duration (s)	0.53	0.29	0.22	<0.01	<0.01	0.29	0.29	0.39	0.99	<0.01	0.81	<0.01
Right Step Duration (s)	0.50	0.71	0.43	0.42	0.72	0.81	0.01	0.72	0.19	0.02	0.06	0.92
Step Duration (s)	0.56	0.38	0.22	0.01	0.02	0.49	0.36	0.51	0.17	0.08	0.37	0.01
Stride Duration (s)	0.49	0.43	0.24	0.01	0.02	0.50	0.34	0.60	0.15	0.08	0.25	0.01
Single Support Time (s)	0.82	0.02	0.15	0.06	0.09	0.01	0.86	0.09	0.05	0.02	0.57	0.05
Double Support Time (s)	<0.01	<0.01	0.63	<0.01	<0.01	0.62	0.47	<0.01	<0.01	0.16	0.18	0.77
Stance Time (s)	0.08	0.54	0.52	<0.01	0.04	0.86	0.21	0.07	<0.01	0.96	0.03	0.06
Swing Time (s)	0.03	<0.01	<0.01	<0.01	0.83	0.01	0.60	0.01	<0.01	<0.01	<0.01	0.91
Single Support (%)	0.15	0.15	0.62	0.01	0.36	0.03	0.56	<0.01	<0.01	0.10	0.07	0.63
Double Support (%)	<0.01	0.10	0.71	<0.01	0.02	0.38	0.23	<0.01	<0.01	0.05	0.35	0.53
Stance Phase (%)	<0.01	<0.01	0.03	<0.01	0.01	0.16	0.12	<0.01	<0.01	<0.01	<0.01	0.02
Swing Phase (%)	<0.01	<0.01	0.03	<0.01	0.01	0.16	0.12	<0.01	<0.01	<0.01	<0.01	0.02
Gait Velocity (m/s)	<0.01	<0.01	0.44	<0.01	<0.01	0.05	0.31	<0.01	<0.01	0.01	<0.01	0.04
Step Length (m)	<0.01	<0.01	0.16	<0.01	<0.01	0.01	0.07	<0.01	<0.01	<0.01	<0.01	0.47
Stride Length (m)	<0.01	<0.01	0.14	<0.01	<0.01	0.02	0.08	<0.01	<0.01	<0.01	0.03	0.75
Step Frequency (steps/min)	<0.01	0.01	0.12	<0.01	<0.01	0.42	0.85	0.18	0.09	<0.01	<0.01	0.10
Walk Ratio (mm/(step/min)	0.02	<0.01	0.06	0.03	<0.01	0.01	0.07	0.11	<0.01	<0.01	0.02	0.15

**Table 9:** *p*-values of the estimated results for Turns with statistically significant *p* < 0.01 for three combinations of participants’ groups (S/EL, S/PD, EL/PD) and for each different test.

Type of Test	WST Slow			WST Normal			WST High			TUG		
Group of Participants	S/EL	S/PD	EL/PD	S/EL	S/PD	EL/PD	S/EL	S/PD	EL/PD	S/EL	S/PD	EL/PD
Number of Recordings	26/18	26/7	18/7	26/18	26/7	18/7	26/18	26/7	18/7	26/18	26/7	18/7
Left Step Duration (s)	0.72	0.97	0.85	0.90	0.66	0.59	0.55	0.44	0.75	0.01	0.64	0.04
Right Step Duration (s)	0.35	0.57	0.36	0.47	0.34	0.10	0.08	0.21	0.98	0.24	0.13	0.33
Step Duration (s)	0.30	0.21	0.11	0.96	0.55	0.59	0.36	0.32	0.09	0.28	0.18	0.18
Stride Duration (s)	0.34	0.66	0.35	0.51	0.60	0.19	0.43	0.77	0.81	0.08	0.17	0.11
Steps Number	<0.01	0.71	0.14	0.94	0.59	0.65	0.08	0.77	0.41	<0.01	0.38	0.09
Single Support Time (s)	0.02	0.55	0.34	0.54	0.84	0.50	0.94	0.17	0.16	<0.01	0.12	0.70
Double Support Time (s)	0.03	0.97	0.17	0.06	0.79	0.24	0.92	0.07	0.06	0.19	0.07	0.21
Stance Time (s)	0.24	0.01	0.05	<0.01	0.79	0.07	0.53	0.50	0.20	0.01	0.87	0.35
Swing Time (s)	0.95	0.20	0.20	0.06	0.49	0.53	0.73	0.16	0.05	<0.01	0.05	0.22
Single Support (%)	0.05	0.88	0.15	0.10	0.71	0.09	0.87	0.45	0.50	<0.01	0.01	0.25
Double Support (%)	<0.01	0.16	0.44	<0.01	0.58	0.02	0.92	0.03	0.01	0.00	0.03	0.56
Stance Phase (%)	<0.01	0.16	0.44	<0.01	0.58	0.02	0.92	0.03	0.01	0.00	0.03	0.56
Swing Phase (%)	0.10	0.08	0.43	0.27	0.39	0.83	0.89	0.87	0.86	0.31	0.13	0.08
Gait Velocity (m/s)	0.73	0.03	0.07	0.80	0.07	0.14	0.14	0.74	0.47	0.27	0.14	0.60
Step Length (m)	0.25	0.01	0.12	0.73	0.15	0.30	0.34	0.76	0.74	0.73	0.25	0.44
Stride Length (m)	0.41	0.11	0.10	0.95	0.68	0.68	0.57	0.15	0.01	0.19	0.70	0.21
Step Frequency (steps/min)	0.63	0.07	0.04	0.99	0.42	0.48	0.14	0.70	0.08	0.26	0.94	0.51
Walk Ratio (mm/(step/min)	0.72	0.97	0.85	0.90	0.66	0.59	0.55	0.44	0.75	0.01	0.64	0.04

## Data Availability

The public version of the Smart-Insole Dataset presented in this study (which includes the raw and annotated data from the insole sensors) is available upon request (Biomedical Informatics Laboratory: https://bmi.hmu.gr, accessed on 10 February 2021 or email at: bmi@hmu.gr) for non-commercial, research and educational purposes only, after the sign of a database usage agreement which establish the terms and conditions of data usage.
